# Changing character and waning impact of COVID-19 at a tertiary centre in Cape Town, South Africa

**DOI:** 10.4102/sajid.v38i1.550

**Published:** 2023-12-18

**Authors:** Lucas E. Hermans, Petro Booysen, Linda Boloko, Marguerite Adriaanse, Timothy J. de Wet, Aimee R. Lifson, Naweed Wadee, Nectarios Papavarnavas, Gert Marais, Nei-yuan Hsiao, Michael-Jon Rosslee, Gregory Symons, Gregory L. Calligaro, Arash Iranzadeh, Robert J. Wilkinson, Ntobeko A.B. Ntusi, Carolyn Williamson, Mary-Ann Davies, Graeme Meintjes, Sean Wasserman

**Affiliations:** 1 Department of Medicine, Faculty of Health Sciences, University of Cape Town, Cape Town, South Africa; 2 Centre for Infectious Diseases Research in Africa, Institute of Infectious Disease and Molecular Medicine, University of Cape Town, Cape Town, South Africa; 3 Division of Infectious Diseases and HIV Medicine, Department of Medicine, University of Cape Town, Cape Town, South Africa; 4 Department of Medical Microbiology, Faculty of Health Sciences, University of Cape town, Cape Town, South Africa; 5 Department of Medicine, Faculty of Internal Medicine, Groote Schuur Hospital, Cape Town, South Africa; 6 Institute of Infectious Disease and HIV Medicine, Department of Medicine, University of Cape Town, Cape Town, South Africa; 7 Division of Medical Virology, Faculty of Health Sciences, University of Cape Town, Cape Town, South Africa; 8 Division of Medical Microbiology, Faculty of Health Sciences, University of Cape Town, Cape Town, South Africa; 9 Department of Medicine, Victoria Hospital, Wynberg, Cape Town, South Africa; 10 Department of Medicine, Division of Pulmonology, Groote Schuur Hospital, Cape Town, South Africa; 11 Department of Integrative Biomedical Sciences, Computational Biology Division, University of Cape Town, Cape Town, South Africa; 12 The Francis Crick Institute, London, United Kingdom; 13 Department of Infectious Disease, Imperial College, London, United Kingdom; 14 South African Medical Research Council, University of Cape Town Extramural Research Unit on the Intersection of Noncommunicable Diseases and Infectious Diseases, Cape Town, South Africa; 15 Department of Pathology, IDM and CIDRI-Africa, Division of Medical Virology, University of Cape Town, Cape Town, South Africa; 16 Department of Health and Wellness, Western Cape Government, Cape Town, South Africa; 17 Centre for Infectious Disease Epidemiology and Research, School of Public Health, University of Cape Town, Cape Town, South Africa; 18 Institute for Infection and Immunity, St George’s, University of London, London, United Kingdom

**Keywords:** SARS-CoV-2, COVID-19, Omicron, Delta, clinical characteristics, observational study

## Abstract

**Background:**

The emergence of genetic variants of SARS-CoV-2 was associated with changing epidemiological characteristics throughout coronavirus disease 2019 (COVID-19) pandemic in population-based studies. Individual-level data on the clinical characteristics of infection with different SARS-CoV-2 variants in African countries is less well documented.

**Objectives:**

To describe the evolving clinical differences observed with the various SARS-CoV-2 variants of concern and compare the Omicron-driven wave in infections to the previous Delta-driven wave.

**Method:**

We performed a retrospective observational cohort study among patients admitted to a South African referral hospital with COVID-19 pneumonia. Patients were stratified by epidemiological wave period, and in a subset, the variants associated with each wave were confirmed by genomic sequencing. Outcomes were analysed by Cox proportional hazard models.

**Results:**

We included 1689 patients were included, representing infection waves driven predominantly by ancestral, Beta, Delta and Omicron BA1/BA2 & BA4/BA5 variants. Crude 28-day mortality was 25.8% (34/133) in the Omicron wave period versus 37.1% (138/374) in the Delta wave period (hazard ratio [HR] 0.68 [95% CI 0.47–1.00] *p* = 0.049); this effect persisted after adjustment for age, gender, HIV status and presence of cardiovascular disease (adjusted HR [aHR] 0.43 [95% CI 0.28–0.67] *p* < 0.001). Hospital-wide SARS-CoV-2 admissions and deaths were highest during the Delta wave period, with a decoupling of SARS-CoV-2 deaths and overall deaths thereafter.

**Conclusion:**

There was lower in-hospital mortality during Omicron-driven waves compared with the prior Delta wave, despite patients admitted during the Omicron wave being at higher risk.

**Contribution:**

This study summarises clinical characteristics associated with SARS-CoV-2 variants during the COVID-19 pandemic at a South African tertiary hospital, demonstrating a waning impact of COVID-19 on healthcare services over time despite epidemic waves driven by new variants. Findings suggest the absence of increasing virulence from later variants and protection from population and individual-level immunity.

## Introduction

The first case of coronavirus disease 2019 (COVID-19) in South Africa was identified in March 2020, and by May 2022 the country had just over four million confirmed cases with 537 000 hospital admissions and more than 100 000 deaths directly attributed over four epidemic waves.^1^

As in other settings, the initial phase of the pandemic was characterised by epidemic waves driven by specific SARS-CoV-2 variants, placing enormous burden on healthcare systems. Waves one, two and three were predominantly driven by Ancestral, Beta and Delta variants, respectively.^2^ Subsequently, after November 2021, the SARS-CoV-2 Omicron variant dominated, and South Africa experienced a fourth wave followed by a fifth resurgence driven by the Omicron BA1/BA2 and BA4/BA5 variants.^3^ Despite the increase in confirmed infections, the ratio of hospital admissions and deaths to infections was markedly lower during the Omicron wave compared with previous waves. This decoupling effect is thought to be because of protective immunity from previous exposure and increasing vaccination coverage;^4^ however, it is not established whether reduced severity is related to attenuation of viral virulence or acquisition of broad host immunity. There are limited studies comparing individual-level characteristics and outcomes by variant, which may enable adjustment for important clinical confounders that are not captured by population-based studies and may help to identify risk factors for more severe COVID-19 as the pandemic evolves.

We undertook a 2-year descriptive analysis of COVID-19 admissions at a referral hospital in South Africa in an attempt to describe the evolving differences between the SARS-CoV-2 variants in clinical phenotypes, individual level characteristics, severity and outcomes, with a focus on the Omicron versus Delta wave periods.

## Methods

### Study design

We performed a retrospective observational cohort study among patients admitted to a South African referral hospital with COVID-19 pneumonia.

### Participants and setting

We identified patients 18 years or older admitted to Groote Schuur Hospital, a large referral hospital in Cape Town, South Africa, with COVID-19 pneumonia, defined as respiratory illness caused by SARS-CoV-2, as confirmed by reverse transcriptase polymerase chain reaction (RT-PCR) or antigen testing on a respiratory sample and requiring supplemental oxygen. For the fourth wave and fifth wave periods, we also included patients admitted with a clinical diagnosis of COVID-19 pneumonia without a positive COVID-19 test result at our facility, if a positive antigen test was documented at another facility or at home, to account for the increased uptake of antigen testing in outpatient settings during this time period. We excluded patients admitted to hospital for reasons not including COVID-19 pneumonia or complications thereof, despite having positive COVID-19 tests.

Patients were included from five distinct periods: first wave, 26 March to 10 July 2020; second wave, 15 November 2020 to 15 January 2021; third wave, 20 May to 16 October 2021; fourth wave, 12 November 2021 to 28 January 2022 and the fifth wave, 23 April 2022 to 31 May 2022, in accordance with definitions of the National Institute of Communicable Diseases (NICD).^5^ These dates were selected to represent a complete time profile of each wave (different phases of the wave period may influence hospital outcomes) and to coincide with the period at which point various variants were dominating in South Africa.

### Data sources and variables

Patient records were captured based on a list with all the consecutive COVID-19 pneumonia hospital admissions during each study period for the first three waves. This complete list was submitted to the hospital records department to request the folders and subsequently captured as folders became available. All the COVID-19 pneumonia admissions were captured during the fourth wave and fifth resurgence.

We collected baseline characteristics including demographics, symptom type and duration and comorbidities. Routine bedside and laboratory investigations of potential prognostic importance were captured. We also extracted data on vaccination status, prescribed medication, oxygen requirements, including use of high flow nasal oxygen (HFNO) and ICU admission. This was done by reviewing written medical records, electronic notes and discharge summaries as documented by treating physicians, attained during consultation with patients or their next-of-kin during admission to hospital. Total weekly COVID-19 and non-COVID-19 admissions and death statistics were provided by the Groote Schuur Hospital Information Management department.

The management of COVID-19 was standardised in Groote Schuur hospital. Corticosteroid use, mainly prednisone 40 mg daily, was introduced to management protocols for all patients with COVID-19 pneumonia on 16 June 2020 after publication of the RECOVERY trial results.^6^ Although some patients initially received intravenous dexamethasone, most were treated with oral prednisone as oral dexamethasone is not available in South Africa. Low molecular weight heparin was provided to all patients at prophylactic doses (0.5 mg/kg daily), in line with hospital guidelines. No patients received remdesivir, IL-6 inhibitors, JAK kinase inhibitors, monoclonal antibodies or targeted antivirals. Allocation of intensive care unit (ICU) beds and high flow oxygen was based on a triage scoring tool developed by the provincial Department of Health^7^ and resource availability.

The partial pressure of arterial oxygen (PaO_2_)/inspired oxygen fraction (PaO_2_/FiO_2_ ratio) at admission was calculated. Discharge from hospital or death during the index admission was ascertained directly from medical records or via the electronic hospital clinical management system. Chronic kidney disease (CKD) was defined as estimated glomerular filtration rate (eGFR) of < 60 mL/min/1.73 m^2^ on admission, plus on another time point at least 3 months previously or when the medical records contained a confirmed diagnosis of renal impairment. Data were captured from medical records directly onto electronic case report forms designed specifically for this study and exported to statistical analysis software. We obtained total COVID-19 admission numbers from the Provincial Health Data Centre, a database repository used by the Western Cape Provincial Department of Health that integrates electronic data from multiple data sources including laboratory results and clinical episodes at provincial facilities and hospitals^8^ and used data generated by the Information Management Department at Groote Schuur hospital.

### SARS-CoV-2 testing and sequencing

All SARS-CoV-2 RT-PCR testing at Groote Schuur and its public sector referral hospitals was done by the on-site National Health Laboratory Services (NHLS) diagnostic laboratory. We also included patients initially tested in private laboratories prior to presentation at Groote Schuur. For RT-PCR testing, three different kits were used, namely the Allplex™ 2019-nCoV assay (Seegene, South Korea), the Abbott RealTime SARS-CoV-2 assay, (Abbott, United States [US]), the Alinity m SARS-CoV-2 assay (Abbott, US) and the Xpert^®^ Xpress SARS-CoV-2 (Cepheid). Cycle threshold (Ct) cut offs differed depending on the assay used; generally a value ≤ 40 in any target gene was considered positive; however, positive test results were often called at the discretion of pathologist, depending on internal control values and specific run performances. For antigen testing, the Panbio™ COVID-19 Ag Rapid Test (Abbott Laboratories, US) kit was used.

To establish the prevalence of viral variants in each wave, we performed whole-genome sequencing of representative samples from each wave group. This was done by randomly selecting positive SARS-CoV-2 PCR samples with a cycle threshold of < 35 and were available from storage at the University of Cape Town and NHLS Division of Medical Virology. No genetic sequencing was done on the first wave samples as there were no reported variants of concern (VOC) until October 2020, and these were therefore presumed to be the ancestral Wuhan strain.^4^ Sequence methodology is described in supplementary material (Appendix 1).

### Statistical analysis

The primary outcome measure was the proportion of patients experiencing in-hospital mortality. The exposure variable was the Delta versus Omicron variant of SARS-CoV-II, which was operationalised as patients presenting during the third wave versus the fourth wave and fifth resurgence, which functioned as proxies for the Delta and Omicron variants, respectively. Clinical practice changed substantially after the first and second wave periods because of rapidly developing clinical guidelines, particularly around corticosteroid use, and the implementation of specific patient referral pathways and upscaling of HFNO and ICU access. We therefore included the first two waves in the descriptive part of the analysis but did not include them in comparative analyses, thus limiting potential bias. A separate comparative analysis between the Wuhan-driven first wave and Beta-driven second wave using this dataset was previously undertaken.^9^

Baseline characteristics are presented as proportions for categorical variables and medians with interquartile range for continuous variables and compared by wave using the Fisher exact test and rank sum test, respectively. We performed survival analysis using Cox proportional hazards models to compare overall mortality between the Delta and Omicron wave periods. The analysis was adjusted for demographic and clinical covariables that were expected to be of relevance for the outcome, namely gender, age, HIV infection, hypertension, diabetes mellitus, CKD and vaccination status. Analysis was performed in R version 4.0.1. Formal power calculation was not performed for this study, as the analysis was exploratory in nature, and the event rates were unknown at the start of the study.

## Ethical considerations

This study was approved by the University of Cape Town Human Research Ethics Committee, study approval number HREC 285/2020. A waiver of consent was granted to obtain and analyse information on disease severity; verbal agreement was documented in the clinical notes for prospective data collection. We maintained the confidentiality of data by ensuring all information was anonymised and we deidentified all PCR samples sent for sequencing.

## Results

### Clinical and viral characteristics

A total of 1689 patients were included: 571 during the first wave period, 611 from the second wave, 374 from the third wave, 118 from the fourth wave and 15 from the fifth resurgence; the latter two periods totalling 133 patients representing the biphasic wave from Omicron variant infections. All patients required supplemental oxygen, with median PaO_2_/FiO_2_ 169 kPa (Interquartile range [IQR] 97–259) over all periods. Evidence of systemic inflammation was present, with a median C-reactive protein (CRP) value of 100 mg/L (IQR 47–168) and D-dimer of 0.56 ug/mL (IQR 0.34–1.02) (Table 1). Cough and dyspnoea were the most frequently reported symptoms and increased in prevalence over time. Cough increased from 66.5% in the first wave to 84.0% in the Omicron wave periods. Dyspnoea went from 73.7% to 88.5% in the same period. In contrast, anosmia was less frequently reported during later waves (from 20.9% during the second (Beta) wave period to 6.9% in the Omicron wave periods. Corticosteroid therapy and anticoagulation prophylaxis were prescribed in over 80% of cases from wave two onwards. The use of HFNO was initially low because of limited availability in the first two waves but scaled up during the third (Delta) wave, followed by a large reduction during the latter stages of the omicron periods (Table 1).

**TABLE 1:** Baseline characteristics.

	Total	Wuhan Strain	Beta	Delta	Omicron	*p*-value (Delta vs Omicron)
***n***	**1689**	**571**	**611**	**374**	**133**	-
**Patient characteristics**						
Sex, Male (%)	761 (45.1)	241 (42.2)	281 (46.0)	174 (46.5)	65 (48.9)	0.715
Age, median (IQR]	55.0 [44.2, 66.0]	53.0 (41.0, 65.0]	58.0 [47.5, 68.0]	52.8 [43.9, 62.1]	59.5 [47.5, 71.4]	< 0.001
Symptoms						
Cough (%)	1271 (75.6)	380 (66.5)	458 (75.0)	323 (87.5)	110 (84.0)	0.379
Dyspnoea (%)	1432 (85.1)	421 (73.7)	549 (89.9)	346 (93.8)	116 (88.5)	0.081
Fever (%)	731 (43.5)	264 (46.2)	248 (40.6)	163 (44.2)	56 (42.7)	0.857
Anosmia (%)	261 (15.5)	65 (11.4)	128 (20.9)	59 (16.0)	9 (6.9)	0.014
Myalgia (%)	459 (27.3)	143 (25.0)	149 (24.4)	126 (34.1)	41 (31.3)	0.627
Comorbidities						
Hypertension (%)	873 (51.7)	279 (48.9)	335 (54.8)	181 (48.4)	78 (58.6)	0.054
Diabetes Mellitus (%)	667 (39.5)	199 (34.9)	273 (44.7)	143 (38.2)	52 (39.1)	0.943
CKD (%)	173 (10.2)	43 (7.5)	66 (10.8)	35 (9.4)	29 (21.8)	< 0.001
HIV infection (%)	169 (11.3)	83 (14.5)	34 (8.2)	30 (8.0)	27 (16.5)	0.009
Vaccination status						< 0.001
Unvaccinated	1405 (83.2)	571 (100.0)	611 (100.0)	149 (39.8)	74 (55.6)	-
Vaccinated	40 (2.4)	0 (0.0)	0 (0.0)	17 (4.5)	23 (17.3)	-
Status not recorded	244 (14.4)	0 (0.0)	0 (0.0)	208 (55.6)	36 (27.1)	-
CRP (mg/L) [IQR]	100 [47, 168]	93 [43,182]	100 [51, 149]	109 [65, 183]	90 [30, 196]	0.224
D-dimer (mg/L) [IQR]	0.56 [0.34, 1.02]	0.66 [0.35, 1.35]	0.50 [0.33, 0.90]	0.55 [0.34, 1.00]	0.76 [0.47, 1.83]	0.029
PF ratio [IQR]	169 [97, 259]	NA [NA, NA]	200 [122, 281]	111 [79, 202]	172 [116, 244]	< 0.001
**Inpatient management and outcomes**						
Corticosteroids (%)	1103 (65.4)	78 (13.7)	566 (92.6)	352 (94.4)	107 (81.7)	< 0.001
Anticoagulation - enoxaparin (%)	1403 (83.2)	358 (62.7)	571 (93.5)	359 (96.2)	115 (87.8)	0.001
Prophylactic enoxaparin dose	570 (55.2)	NA (NA)	218 (38.2)	257 (73.6)	95 (84.8)	0.008
Therapeutic enoxaparin dose	462 (44.8)	NA (NA)	353 (61.8)	92 (26.4)	17 (15.2)	-
High-flow nasal cannula (%)	365 (21.6)	49 (8.6)	94 (15.4)	202 (54.0)	20 (15.3)	< 0.001
ICU admission (%)	120 (7.1)	35 (6.1)	36 (5.9)	45 (12.1)	4 (3.1)	0.005
Deceased (%)	434 (25.7)	127 (22.2)	135 (22.1)	138 (37.1)	34 (25.8)	0.024

Note: *P*-value denotes uni-variable comparisons between delta and omicron variants. CKD, Chronic kidney disease; CRP, C-reactive Protein; HIV, Human Immunodeficiency Virus; PaO_2_/FiO_2_ ratio, partial pressure of arterial oxygen/inspired oxygen fraction.

### SARS-CoV-2 variant determination

Genotyping confirmed known epidemiological associations with wave periods: Beta variant in the second wave (97% [113/117]), Delta variant in the third wave (79.6% [86/108]), Omicron BA.1, BA.1.17, BA.1.17.2, BA.1.18 variants in the fourth wave (95.5% [21/22]) and Omicron BA.4, BA4.1 and BA4.7 variants in the fifth resurgence (100% [3/3]).

### Comparison of clinical characteristics: Delta versus Omicron cohorts

Notable differences in patient characteristics between the Delta cohort (third wave) and Omicron cohort (fourth wave and fifth resurgence) included older age in the Omicron cohort (59.5 years vs. 52.8 years; *p* < 0.0001) and higher prevalence of HIV (16.5% vs. 8.0%; *p* = 0.009), CKD (21.8% vs. 9.4%; *p* < 0.001) and hypertension (58.6% vs. 48.4%; *p* = 0.054) in the Omicron cohort compared to the Delta cohort. While the proportion of vaccinated patients increased over time (4.5% in Delta cohort vs. 17.3% in Omicron cohort; *p* < 0.001), most patients were unvaccinated. Severity of illness was significantly lower in the Omicron cohort, as evidenced by higher PaO_2_/FiO_2_ ratios in the Omicron versus Delta cohort (172 vs. 111; *p* < 0.001). Significantly fewer patients in the Omicron cohort received HFNO (15.3% vs. 54.0%; *p* < 0.001) or were admitted to ICU for invasive mechanical ventilation (3.1% vs. 12.1%; *p* < 0.001) compared with patients in the Delta cohort.

### Mortality

Crude in-hospital mortality was lower in the Omicron compared to Delta wave period (25.8% [34/133] vs. 37.1% [138/374]; HR 0.68 [95% CI 0.47–1.00] *p* = 0.049); this effect persisted after adjustment for age, sex, HIV status and other comorbidities (aHR 0.43 [95% CI 0.28–0.67] *p* < 0.001) (Table 2).

**TABLE 2:** Factors associated with in-hospital mortality.

Covariable	In-hospital mortality		Multivariable Cox model		
	Deceased (*n* = 172)	Alive (*n* = 332)	aHR	95% confidence interval	*p*-value
Viral variant: - *Omicron*	34 (19.8%)	98 (29.5%)	0.43	028–0.67	< 0.001
Seat - *Male*	83 (48.3%)	154 (46.4%)	1.14	084–1.54	0.415
Age	58.6 [48.2–6 6.2]	52.7(42.5–62.3)	1.03	1.01–1.04	< 0.001
HIV infection	23 (13.4%)	29 (8.7%)	1.81	1.14–2.88	0.012
Hypertension	100 (58.1%)	159 (47.9%)	0.97	0.69–1.37	0.873
Diabetes mellitus	76 (44.2%)	119 (35.8%)	1.25	0.91–1.71	0.162
Chronic kidney disease	33 (19.2%)	31 (93%)	1.84	1.18–2.87	0.007
**Vaccination status**					
Unvaccinated	70 (40.7%)	151 (45.5%)	Ref	Ref	Ref
Vaccinated	11 (6.4%)	28 (8.4%)	0.80	0.41–1.55	0.508
Status not recorded	91 (52.9%)	153 (46.1%)	0.92	0.67–1.28	0.636

Cox proportional hazards analysis of risk factors for mortality at 28 days after admission. Cardiovascular disease is a composite variable consisting of hypertension, diabetes, and renal disease. aHR, adjusted Hazard Ratio; Ref, Reference category.

### Hospital admission and death trends over time

The hospital-wide number of admitted patients testing positive for SARS-CoV-2, as well as the number of deaths with SARS-CoV-2, were highest during the third wave dominated by the Delta variant (Figure 1). Graphical representation of weekly hospital level deaths, stratified by SARS-CoV-2 positive and overall reveals a gradual decoupling of SARS-CoV-2 deaths and overall deaths over time, with overall hospital deaths remaining stable between the onset of the fourth wave in November 2021 until the end of the fifth resurgence in June 2022 (Figure 2).

**FIGURE 1:**
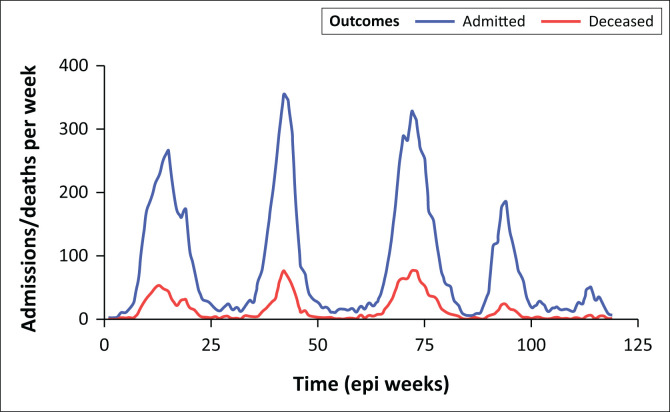
COVID-19 admissions and deaths at Groote Schuur Hospital over time.

**FIGURE 2:**
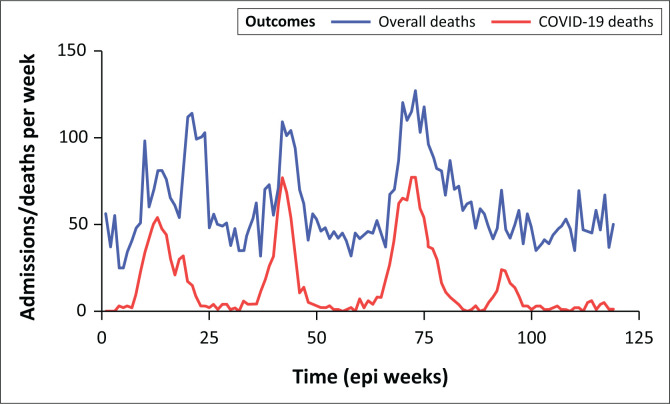
COVID-19 and non-COVID-19 deaths at Groote Schuur Hospital over time.

### Sensitivity and supplementary analyses

The univariable comparison between the Delta and Omicron cohorts was repeated in a sensitivity analysis of unvaccinated patients only, yielding similar results (Appendix 2).

## Discussion

The clinical phenotype and severity of SARS-CoV-2 and its impact on overall hospital mortality and admissions changed markedly over time. Disease severity and inpatient mortality during infection with Omicron variants were substantially lower compared with the Delta variant, even though patients admitted during the Omicron waves had more comorbidities and were older.

Early epidemiological signals from South Africa suggested that Omicron-infected patients were less likely to develop severe disease,^3,10,11^ which was later confirmed by formal studies comparing disease outcomes of Omicron to other VOC.^4^ Our study replicated these observations, showing that the Omicron cohort had less severe respiratory disease, with lower oxygen requirements and higher PaO_2_/FiO_2_ ratios when compared to the Delta cohort, resulting in significantly fewer patients requiring HFNO or ICU admission, despite the hospital’s increased capacity to accommodate severely ill patients during this period.^12^ These findings are in line with studies performed in settings outside of South Africa, which have also shown decreased disease severity from Omicron infections compared to previous variants.^11^

The reduced disease severity is likely because of a combination of the reduced pathogenic potential of the Omicron variant, which is less effective at replicating in lung parenchyma when compared to previous variants,^13,14^ and increased rates of vaccination and previous exposure during the Omicron period.^4^ Our data showed that patients in the Omicron cohort had more frequently been vaccinated against COVID-19, especially those admitted during the fifth resurgence, which likely reflects the rapid growth of the vaccinated population during this timeframe rather than weaning vaccine efficacy in admitted patients. Lack of apparent vaccine efficacy in our cohort is likely because of low patient numbers (with poor precision around estimates) and the presence of protective natural immunity from prior infection.

Although population-based studies showed that the mean age of patients presenting to emergency departments in South Africa with confirmed SARS-CoV-2 during the Omicron wave was much lower compared to previous waves,^15^ our data reflect a higher median age in the Omicron cohort. This trend is likely explained by our study population, which included only inpatients, and suggests that young patients presenting to healthcare facility were mostly being discharged home, while patients requiring admission during infection were older. Increasing age is associated with declining kidney function and possibly explains the higher prevalence of CKD in our Omicron cohort, together with a higher propensity for admission among people with chronic medical conditions.^16^ Our study also found that HIV was associated with increased mortality risk in the Omicron cohort, a finding consistent with previous observations.^17^

From a health systems perspective, we demonstrate how the emergence of the Omicron variant coincided with a decoupling between admitted patients testing positive for SARS-CoV-2 and the number of overall hospital deaths. In response to the COVID-19 pandemic, Groote Schuur Hospital implemented several novel measures to deal with the surge in patient load, specifically with improved referral pathways, HFNO management in wards, streamlined ICU referrals and standardised triage scoring systems.^12^ Expertise in the management of COVID-19, acquired over time by hospital staff, would also have contributed to the reduced number of deaths, especially when coupled with a decrease in hospital admissions and demands on the overall healthcare system. During the Omicron-driven fourth wave and fifth resurgence, the overall mortality rate in the hospital was largely constant. This finding underscores that, while COVID-19 incidence during the fourth wave was higher compared to previous waves in South Africa,^4^ the effect of these infections on hospital-wide morbidity and mortality was markedly reduced.

Limitations of our study include retrospective medical record review potentially causing data quality issues and/or missing data. Specifically, during the third wave, many requested patient folders were not readily available from the records department. This may have led to selection bias from inconsistent record retrieval. Groote Schuur Hospital is a referral centre and may not reflect the general population, limiting generalisability. Some missing data points, like laboratory values, were missing at random as certain blood tests might not have been requested on admission day or rejected by laboratories. This did not result in bias in analysis as these parameters were not added to multivariable models. Vaccination status was obtained from medical notes, and many records did not specify full vaccination history. In order to mitigate the risk of missing vaccination status data as a confounder in the multivariable analysis, missing vaccination data were entered as a separate categorical value in the multivariable analysis. Despite adjustment for important clinical confounders, our analysis is not able to establish whether variants independently influence mortality risk because each wave period is dominated by single variant, and there are powerful temporal trends that influence outcome, including population-level immunity and improved clinical and health system management.

## Conclusion

This study confirms prior observations of changing clinical manifestations and lower disease severity and mortality despite ongoing high community transmission and demonstrates a decoupling between COVID-19 admissions and hospital deaths during the biphasic Omicron-driven wave. Viral respiratory infections remain a frequent finding in admitted patients and continued surveillance of the incidence and pathological potential of these infections in the South African setting is of vital importance.

## Figures and Tables

**FIGURE 1-A2:**
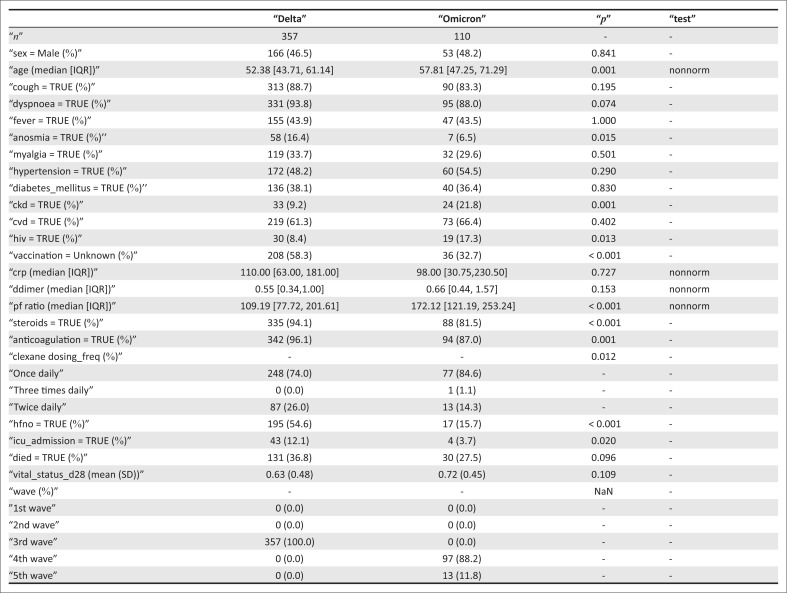
Comparison of Delta and Omicron waves in unvaccinated individuals only.

## Appendix 1: Sequencing method

Briefly, RNA was extracted from nasopharyngeal swabs of qPCR-confirmed COVID-19 patients using automated methods. Complementary DNA was synthesised using the Superscript IV reverse transcriptase (Life Technologies, Carlsbad, CA) and random hexamer primers. SARS-CoV-2 whole genome amplification was performed using the ARTIC V3 protocol.^1^

Below follows a brief summary of how multiplex PCR was performed using primers designed on Primal Scheme (http://primal.zibraproject.org/) to generate 400 base pair (bp) amplicons with a 70 bp overlap covering the SARS-CoV-2 genome. PCR products were purified using AMPure XP magnetic beads (Beckman Coulter, CA) and quantified using the Qubit dsDNA High Sensitivity assay on the Qubit 3.0 instrument (Life Technologies Carlsbad, CA). The Illumina^®^ DNA Prep kit with Nextera DNA CD Indexes (96 indexes) was used to prepare indexed paired-end libraries of genomic DNA. Sequencing libraries were normalised to 4 nM, pooled and denatured with 0.2 N sodium hydroxide. Libraries were sequenced on the Illumina MiSeq instrument (Illumina, San Diego, CA, USA). Paired-end fastq reads were assembled using Genome Detective 1.132 (https://www.genomedetective.com) and the Coronavirus Typing Tool. The assembly obtained from Genome Detective were submitted to the Nextclade webpage (https://clades.nextstrain.org) for the initial quality assessment. Genomes were polished by aligning mapped reads to the references and filtering out low-quality mutations using bcftools 1.7–2 mpileup method. Mutations were confirmed visually with bam files using Geneious software (Biomatters Ltd, New Zealand). Any assembled Genomes with missing sites greater than 10% of the reference genome size were removed from the dataset (> 3000 base pairs). To assign the sequenced samples to their lineage and clade, we used the dynamic lineage classification method proposed by Rambault et al. via the Phylogenetic Assignment of named Global Outbreak LINeages (PANGOLIN) software suite (https://github.com/hCoV2019/pangolin) 4, and Nextclade5, respectively.
